# Dietary total antioxidant capacity in relation to metabolic health status in overweight and obese adolescents

**DOI:** 10.1186/s12937-022-00806-9

**Published:** 2022-08-30

**Authors:** Sobhan Mohammadi, Keyhan Lotfi, Saeideh Mirzaei, Ali Asadi, Masoumeh Akhlaghi, Parvane Saneei

**Affiliations:** 1 Department of Community Nutrition, School of Nutrition and Food Science, Nutrition and Food Security Research Center, Students’ Research Committee, Isfahan University of Medical Sciences, Isfahan, Iran; 2 Department of Community Nutrition, School of Nutritional Sciences and Dietetics, Tehran University of Medical Sciences, Tehran, Iran; 3 Department of Community Nutrition, School of Nutrition and Food Science, Shiraz University of Medical Sciences, Shiraz, Iran; 4 Department of Exercise Physiology, School of Physical Education and Sport Sciences, University of Tehran, Tehran, Iran; 5 Department of Community Nutrition, School of Nutrition and Food Sciences, Shiraz University of Medical Sciences, Shiraz, Iran; 6 Department of Community Nutrition, School of Nutrition and Food Science, Nutrition and Food Security Research Center, Isfahan University of Medical Sciences, PO Box 81745-151, Isfahan, Iran

**Keywords:** Dietary total antioxidant capacity, Obesity, Metabolically health status, Adolescents, Children

## Abstract

**Background:**

Although several studies evaluated the relationship between individual dietary antioxidants and metabolic health conditions, data on the association between dietary total antioxidant capacity and metabolic health among children and adolescents is limited. This study investigated the relationship between dietary total antioxidant capacity and metabolic health status in Iranian overweight/obese adolescents.

**Methods:**

This cross-sectional study was conducted on 203 overweight/obese adolescents. Dietary intakes were evaluated by a validated food frequency questionnaire. Ferric Reducing-Antioxidant Power (FRAP) was considered to indicate dietary total antioxidant capacity. Anthropometric parameters and blood pressure status were measured. Fasting blood samples were obtained to determine circulating insulin, glucose, and lipid profile. Two different methods (modified International Diabetes Federation (IDF) criteria and IDF criteria along with insulin resistance) were applied to classify participants as metabolically healthy obese (MHO) or metabolically unhealthy obese (MUO).

**Results:**

According to IDF and IDF/HOMA definitions, a total of 79 (38.9%) and 67 (33.0%) adolescents were respectively defined as MUO. Considering IDF criteria, the highest tertile of FRAP was related to lower odds of being MUO in the maximally-adjusted model (OR: 0.40; 95%CI: 0.16–0.96), compared to the lowest tertile. However, based on the IDF/HOMA-IR criteria, no significant relation was found between FRAP and odds of MUO (OR: 0.49; 95%CI: 0.19–1.23) after considering all possible confounders.

**Conclusions:**

Adolescents with higher intakes of dietary antioxidants have a lower possibility of being MUO based on IDF criteria. However, no substantial relation was found considering HOMA-IR/IDF definition. Further prospective cohort studies need to be done to confirm these findings.

## Introduction

Overweight and obesity among children and adolescents have recently become a main health concern worldwide [1]. Obesity is considered a primary cause of various metabolic disorders development [2]. A wide range of long-standing health complications might arise as a result of metabolic abnormalities in children, as well as an increased risk of all-cause mortality in adulthood [3, 4]. According to estimates, the prevalence of childhood and adolescent obesity has nearly doubled in recent decades [5]. Based on a meta-analysis study, about 16% of Iranian children and adolescents are overweight or obese [6]. It is noteworthy to consider the metabolic status of overweight or obese individuals [7]. Despite having excess body fat, some individuals are known as metabolically healthy obese (MHO), having no metabolic disturbances [8]. Whereas, the concomitant occurrence of obesity and metabolic abnormalities is recognized as metabolically unhealthy obesity (MUO) [9].

Urban development, poor diets, and sedentary lifestyles also raise the prevalence of childhood obesity [10]. According to prior studies, the development of obesity is exacerbated by the overproduction of reactive oxygen species (ROS) [11, 12]. Also, oxidative stress that is caused by obesity might induce chronic inflammation, which is an important factor in the pathophysiology of metabolic abnormalities as well [13]. Dietary antioxidants have been recommended to defend against oxidative stress and inflammatory complications [14, 15]. In some earlier studies, antioxidants such as vitamin C, vitamin E, carotenoids, and selenium levels were inversely related to the risk of overweight and obesity [16, 17]. However, an individual antioxidant or food item may not accurately reflect the total antioxidant capacity of a diet, therefore, dietary total antioxidant capacity (DTAC) has been developed and attracted considerable attention [18]. DTAC has been proposed as a method for studying the potential beneficial impacts of the whole dietary antioxidants and has been found to have a strong correlation with plasma antioxidant capacity [18, 19].

A wide range of studies indicated that plasma TAC levels are improved by consuming antioxidant-rich food sources such as fruits and vegetables [20, 21]. According to several studies, higher DTAC might beneficially affect various metabolic disturbances [20, 22]. Furthermore, diets high in antioxidants could negatively be associated with plasma C-reactive protein (CRP), a major biomarker for systemic inflammation, and in turn, triggering metabolic disorders [23], such as central obesity and glucose intolerance [24]. In addition to glucose abnormality, a cross-sectional study showed that higher DTAC was associated with lower odds of impaired lipid profiles in young participants [24]. Total antioxidant intake was also linked to lower body mass index (BMI) and systolic blood pressure [19]. In addition, decreased TAC of plasma has been associated with metabolic syndrome in young adults [19]. Another cross-sectional study did not confirm this finding; on the other hand, it indicated that plasma TAC in pubertal obese children was higher than normal-weight subjects [25]. Therefore, insufficient data is available on the relationship between DTAC and MHO/MUO. More studies are needed to shed a light on this relation, especially in adolescents. In addition, few studies have been conducted in the Middle East, where dietary intakes are different from ones in Western countries. Due to these limitations, the current cross-sectional study was conducted to examine the relation between DTAC and MUO among overweight and obese Iranian adolescents.

## Methods

### Participants

This cross-sectional study was performed among a sample of 203 Iranian overweight/obese adolescents (102 girls and 101 boys), aged 12–18 years. The sample size of the study was determined based on data from previously published studies [26, 27], estimating that 40–60% of the Iranian overweight and obese adolescents have been recognized as MUO. Thus, considering a power of 80% and type I error of 0.05, desired confidence interval of 0.95, and precision (d) of 7%, the minimum required sample size was estimated to be 188 subjects. Participants were chosen by multistage cluster random sampling method. Students were selected randomly from five major regions of Isfahan, Iran. Based on age- and gender standardized BMI z-scores [28], overweight and obese children were selected to participate in the current investigation from sixteen schools. Using this approach, adolescents from a wide range of social and economic statuses were involved in this study. Participants who were on a weight-loss diet, using vitamin and mineral supplements, or taking drugs that might affect their body weight, blood glucose, lipid profile, or blood pressure were excluded. Furthermore, we did not include students with genetic or endocrine abnormalities (such as type 1 diabetes mellitus, hypothyroidism and Cushing syndrome) in this investigation. Therefore, we enrolled 203 overweight/obese adolescents in the current analysis. The written informed agreement was obtained from each participant. In addition, informed consents were obtained from their parents. The Isfahan University of Medical Sciences local ethics committee was responsible for the study protocol.

### Dietary assessment and calculation of DTAC

A validated 147-item food frequency questionnaire (FFQ) was applied to collect data on the dietary intakes of the individuals [29]. Former investigations showed that this FFQ could be an accurate indicator of dietary intake about various diseases among Iranian adolescents Consumption for each food item was questioned based on a daily, weekly, or monthly frequency. Afterward, using household measurements, portion sizes of consumed food items were converted to grams per day [30]. Then, gram/day values were input into Nutritionist IV software to calculate nutrient intake. The Nutritionist IV software used the USDA food composition database, with some modifications based on Iranian foods. Ferric Reducing-Antioxidant Power (FRAP) values were obtained from a previously published report to estimate DTAC [31]. The FRAP assay measures dietary antioxidants ability to convert ferric ions to ferrous, and is indicated as mmol/100 g of food [32]. Whenever FRAP data was not available for a specific food item, based on its ingredients, the value of the closest equivalent item was allocated by the principal investigator (P.S.). Finally, the frequency of consumption of each food item was multiplied by their related FRAP values. FRAP values were then summed up to compute the DTAC for each participant.

### Assessment of anthropometric indices and cardiometabolic risk factors

Two trained dietitians used a stadiometer to measure standing height without shoes (to the nearest 0.1 cm). Weight was also measured with a calibrated electronic scale to the nearest 0.1 kg in minimal clothing and without shoes. Then, participants were classified as normal, overweight, or obese adolescents based on the age-and sex-standardized BMI z-scores defined by World Health Organization (WHO) for adolescents [28]. After normal respiration and without putting any pressure on the body surface, the waist circumference (WC) value was measured with a flexible tape (to the nearest 0.1 cm). A mercury sphygmomanometer with a suitable cuff size was used to measure systolic blood pressure (SBP) and diastolic blood pressure (DBP) on the right arm, twice after a 15-min recovery time. The average of the two measurements was calculated and used in this study. The concentrations of triglycerides (TG), high-density lipoprotein cholesterol (HDL-c), and fasting blood glucose (FBG) were measured. The Homeostasis Model Insulin Resistance (HOMA-IR) was determined using the following formula to measure Insulin resistance (IR) [33] HOMA-IR = [Fasting glucose (mg/dl) × fasting insulin (μu/ml)]/405.

### Assessment of metabolic status

Two strategies were applied to classify participants as MHO or MUO individuals. The first approach had the same components as metabolic syndrome (MetS) based on the children’s International Diabetes Federation criteria [34]. MUO children were defined as having two or more MetS components such as increased triglycerides (≥ 150 mg/dL), decreased HDL-c (< 40 mg/dL for the age of < 16 y, and < 50 mg/dL in girls/ < 40 mg/dL in boys for the age of ≥ 16 y), increased fasting blood glucose (≥ 100 mg/dL) and elevated blood pressure (≥ 130/85 mmHg). Other than that, they were defined as MHO subjects. In the second method, HOMA-IR was considered along with IDF criteria that were used in the first classification method. Individuals with HOMA-IR score ≥ 3.16 who had at least two other metabolic risk factors were classified as MUO; and those with HOMA-IR < 3.16 were considered as MHO adolescents [9, 35].

### Assessment of other variables

The Physical Activity Questionnaire for Adolescents (PAQ-A) was applied to assess the participants physical activity levels. This questionnaire consists of nine questions on different activities to evaluate physical activity in the last week [36]. Items 1 to 8 of the questionnaire were scored from 1-to 5; a score of 1 indicated the lowest and 5 showed the highest level of physical activity. The ninth question evaluated the unusual activity of adolescents during the previous 7 days. Scores were summed up and adolescents were categorized into very active (score ≥ 4), moderate (4 < score ≤ 3), less active (3 < score ≤ 2), and sedentary or not having an orderly week activity (score < 2). A validated demographic questionnaire was used by trained researchers to evaluate the socioeconomic status (SES) of the students [37] based on the following variables: parental job, the number of family members, parental education level, having cars in the family, having computers/laptops, having personal room and taking trips in the year. Afterward, the SES was computed to have a total score. A questionnaire was used to record the participants’ age, gender, medical history, medication, and supplement use.

### Statistical analysis

FRAP values were adjusted for total energy intake using the residual method to have an independent exposure from the energy intake. Then, the individuals were classified according to tertiles of FRAP (T1: < 6.42; T2: 6.42–8.33; T3: > 8.39 mmol/d). General characteristics of study participants across tertiles of FRAP were reported as means ± SDs for continuous and percentages for categorical variables. To examine the differences across tertiles of FRAP, Analysis of Variance (ANOVA) and Chi-square tests were respectively used for continuous and categorical variables. The age, sex, and energy-adjusted dietary intakes of study participants across tertiles of FRAP were evaluated using Analysis of Covariance (ANCOVA). To identify the association between tertiles of FRAP and MUO, multivariable logistic regression was applied. The odds ratios (OR) and 95% confidence intervals (CI) for MUO were calculated in crude and adjusted models. Energy intake could affect the outcome of interest based on previous investigations [38–40]. In the first model, age, gender, and energy intake were adjusted. In the second model, further adjustments were done for physical activity level and SES. Prior studies have shown that omega-3 could also be associated with metabolic risk factors; so, we considered intake of omega-3 fatty acids as a confounder [41]. In the last model, omega-3 fatty acids intake and BMI were adjusted in addition to the previous confounders. In all models, the first tertile of FRAP was considered as the reference. To evaluate the trend of ORs across tertiles of FRAP, the tertiles were considered as an ordinal variable in the analysis. *P*-values < 0.05 (two-sided) were considered the statistically significant level. SPSS software version 26 (IBM, Chicago, IL) was used for all analyses.

## Results

The mean age of the study population was 13.98 years. According to the BMI category, 51.2% and 48.8% of adolescents were respectively overweight and obese. Based on the first criteria of metabolic health status (IDF), 61.1% (*n* = 124) of the individuals were classified as MHO (64 boys, 60 girls), and 38.9% (*n* = 79) others were MUO (37 boys, 42 girls). While, according to the second definition (HOMA-IR), 67.00% (*n* = 136) of participants were categorized as MHO (66 boys, 70 girls), and 33.00% (*n* = 67) others were MUO (35 boys, 32 girls). General characteristics and cardio-metabolic status of the study participants across tertiles of FRAP are shown in Table 1. Participants in the highest tertile of FRAP, compared to the lowest, had lower DBP (*P* < 0.04) and FBG (*P* < 0.01). No significant differences were observed in terms of age, weight, sex, BMI, physical activity level, SES, SBP, insulin, HOMA-IR, TG, and HDL cholesterol across tertiles of FRAP.

**Table 1:** General characteristics of study participants across energy-adjusted tertiles of the dietary TAC score (*n* = 203)^a^

	Tertiles of energy-adjusted FRAP			P^b^
	T_1_ (*n* = 67)	T_2_ (*n* = 68)	T_3_ (*n* = 68)	
Age (y)	13.84 ± 1.62	13.94 ± 1.50	14.16 ± 1.69	0.49
Weight (kg)	73.66 ± 10.81	73.53 ± 12.85	73.25 ± 11.21	0.98
Body mass index (kg/m^2^)	27.26 ± 2.74	27.35 ± 3.74	27.44 ± 3.20	0.95
Gender (%)				0.32
Boy	44.8	48.5	57.4	
Girl	55.2	51.5	42.6	
Physical activity levels (%)				0.78
Low	53.7	48.5	48.5	
High	46.3	51.5	51.5	
Socioeconomic status levels (%)				0.72
Low	32.8	30.9	23.5	
Moderate	44.8	42.6	45.6	
High	22.4	26.5	30.9	
Systolic blood pressure (mmHg)	115.67 ± 11.41	111.60 ± 16.87	110.88 ± 24.26	0.26
Diastolic blood pressure (mmHg)	75.02 ± 10.03	74.81 ± 6.75	70.68 ± 15.27	0.04
Fasting blood glucose (mg/dL)	100.64 ± 9.71	96.84 ± 7.46	96.46 ± 7.76	0.01
Insulin (µUI/mL)	22.33 ± 15.13	21.00 ± 11.22	17.97 ± 11.02	0.12
HOMA-IR index	5.55 ± 3.72	5.13 ± 3.07	4.38 ± 3.2.94	0.11
Triglycerides (mg/dL)	127.58 ± 63.19	129.82 ± 67.89	108.53 ± 67.35	0.12
HDL cholesterol (mg/dL)	43.67 ± 6.76	44.91 ± 9.05	45.87 ± 7.74	0.27

^a^All values are means ± standard deviation (SD), unless indicated

^b^Obtained from ANOVA for continuous variables and chi-square test for categorical variables

Dietary intakes of adolescents across tertiles of FRAP are presented in Table 2. Subjects in the top tertile of FRAP, compared to those in the bottom tertile, had higher intakes of fruits, vegetables, coffee and tea, nuts, total fat, dietary fiber, selenium, magnesium, vitamin A, beta-carotene, alpha-tocopherol, and vitamin C. Furthermore, they consumed lower amounts of carbohydrate and iron. No significant differences were found in the total energy, meat, protein, and vitamin E intake across tertiles of FRAP.

**Table 2:** Multivariable-adjusted intakes of Dietary TAC Score and selected nutrients of study participants across energy-adjusted tertiles of the dietary TAC score (*n* = 203)^a^

	Tertiles of energy-adjusted FRAP			P^b^
	T_1_ (*n* = 67)	T_2_ (*n* = 68)	T_3_ (*n* = 68)	
Energy (Kcal/d)	2896.43 ± 66.52	2831.28 ± 65.88	2921 ± 66.14	0.61
**Food groups (g/day):**				
Fruits	234.41 ± 17.84	343.75 ± 17.70	417.91 ± 17.75	< 0.001
Vegetables	214.20 ± 20.39	279.39 ± 20.23	333.67 ± 20.29	< 0.001
Meats	68.43 ± 4.02	68.56 ± 3.99	69.05 ± 4.00	0.99
Coffee and Tea	99.69 ± 22.96	237.37 ± 22.77	570.95 ± 22.84	< 0.001
Nuts	7.87 ± 1.28	11.99 ± 1.27	16.61 ± 1.27	< 0.001
**Other nutrients:**				
Proteins (% of energy)	14.05 ± 0.25	14.25 ± 0.24	14.62 ± 0.24	0.25
Fats (% of energy)	27.50 ± 0.62	28.42 ± 0.61	30.60 ± 0.62	< 0.001
Carbohydrates (% of energy)	59.54 ± 0.62	58.79 ± 0.61	56.56 ± 0.62	< 0.001
Dietary fiber (g/d)	16.72 ± 0.54	19.63 ± 0.53	21.95 ± 0.53	< 0.001
Omega-3 fatty acids (g/d)	0.57 ± 0.02	0.60 ± 0.02	0.64 ± 0.02	0.04
Selenium (mg/d)	0.10 ± 0.00	0.08 ± 0.00	0.09 ± 0.00	< 0.001
Iron (mg/d)	26.49 ± 0.64	25.30 ± 0.63	23.03 ± 0.63	< 0.001
Magnesium (mg/d)	255.17 ± 6.72	279.11 ± 6.66	329.79 ± 6.68	< 0.001
Vitamin A (µg/d)	856.09 ± 73.97	1147.80 ± 73.37	1314.69 ± 73.60	< 0.001
Beta-Carotene (µg/d)	280.10 ± 67.42	468.58 ± 66.87	525.69 ± 67.08	0.03
Vitamin E (mg/d)	29.04 ± 1.40	32.20 ± 1.39	29.81 ± 1.40	0.25
Alpha-Tocopherol (mg/d)	10.53 ± 0.59	12.50 ± 0.58	14.30 ± 0.59	< 0.001
Vitamin C (mg/d)	98.80 ± 6.41	136.52 ± 6.36	165.07 ± 6.38	< 0.001

^a^All values are means ± standard error (SE); energy intake and macronutrients are adjusted for age and gender; all other values are adjusted for age, gender and energy intake. ^b^Obtained from ANCOVA

**Fig. 1:**
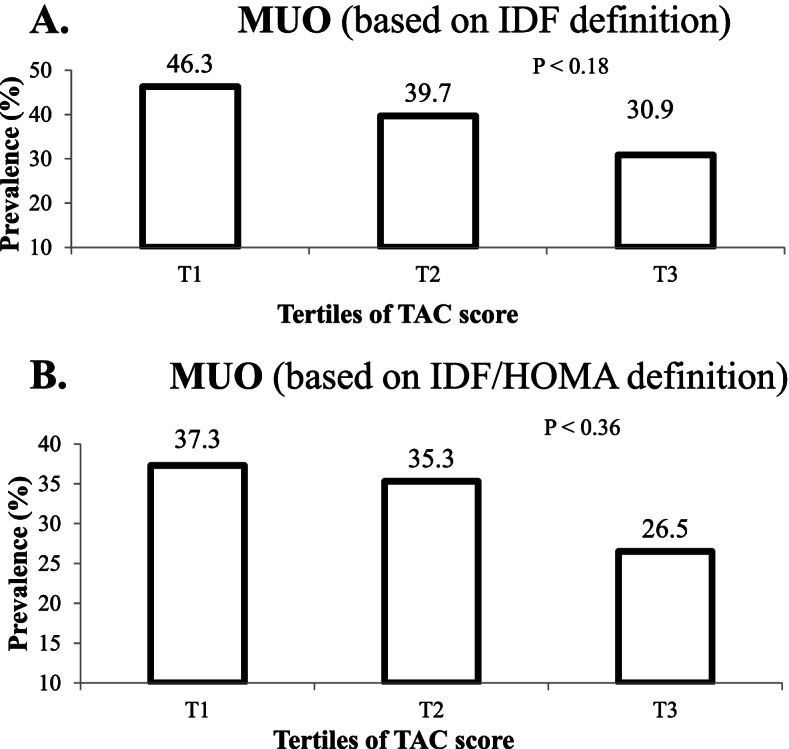
Prevalence of MUO across tertiles of FRAP in the study population **A** MUO based on IDF definition. **B** MUO based on IDF/HOMA-IR definition

Figure 1 illustrates the prevalence of MUO across tertiles of FRAP, considering both IDF and IDF/HOMA-IR definitions. According to the IDF criteria, MUO prevalence in the third tertile of FRAP was not significantly different from the first tertile (30.9 vs. 46.3%, *P* = 0.18). Similarly, based on the IDF/HOMA-IR criteria, individuals in the top tertile of FRAP did not have a substantial difference in the prevalence of MUO, compared to the bottom tertile (26.5 vs. 37.3%, *P* = 0.36).

Multivariable-adjusted odds ratios for MUO, based on both definitions, across tertiles of FRAP are presented in Table 3. Based on the IDF definition, adolescents in the highest tertile of FRAP, compared to the lowest had 48% decreased odds of MUO in the crude model. However, this association was not significant (OR: 0.52; 95% CI: 0.26–1.05; *P* = 0.07). After controlling all potential cofounders, the association became stronger and more significant (OR: 0.40; 95% CI: 0.16–0.96; *P* = 0.04); such that those in the top tertile of FRAP had 60% reduced odds of MUO, compared to the bottom tertile. Considering the IDF/HOMA-IR definition, there was not any significant association between FRAP and MUO in crude (OR for T3 vs. T1: 0.60; 95% CI: 0.29–1.26; *P* = 0.18) and fully-adjusted (OR for T3 vs. T1: 0.47; 95% CI: 0.19–1.19; *P* = 0.11) models.

**Table 3:** Multivariable-adjusted odds ratio for MUO across energy-adjusted tertiles of the dietary TAC score (*n* = 203)^a^

	Tertiles of energy-adjusted FRAP			
	T_1_ (*n* = 67)	T_2_ (*n* = 68)	T_3_ (*n* = 68)	P_trend_
**MUO Based on IDF criteria**				
MUO cases (n)	31	27	21	
Crude	1.00	0.76 (0.39–1.51)	0.52 (0.26–1.05)	0.07
Model 1	1.00	0.82 (0.40–1.66)	0.46 (0.22–0.99)	0.05
Model 2	1.00	0.86 (0.38–1.94)	0.50 (0.21–1.15)	0.11
Model 3	1.00	0.79 (0.35–1.81)	0.40 (0.16–0.96)	0.04
**MUO Based on IDF/HOMA-IR criteria**				
MUO cases (n)	25	24	18	
Crude	1.00	0.92 (0.45–1.85)	0.60 (0.29–1.26)	0.18
Model 1	1.00	1.03 (0.50–2.15)	0.56 (0.25–1.24)	0.17
Model 2	1.00	1.11 (0.48–2.55)	0.61 (0.25–1.47)	0.30
Model 3	1.00	1.04 (0.43–2.36)	0.47 (0.19–1.19)	0.14

^a^All values are odds ratios and 95% confidence intervals. P_trend_ was obtained by the use of tertiles of TAC score as an ordinal variable in the model. Model 1: Adjusted for age, gender, energy intake. Model 2: More adjustments for physical activity levels, socioeconomic status. Model 3: Further adjustment for omega-3 and BMI

## Discussion

In the current cross-sectional study on Iranian adolescents, a significant inverse association was found between FRAP and incidence of being MUO based on the IDF criteria for MUO. This association was independent of potential confounders. However, following IDF/HOMA-IR criteria, FRAP levels were not substantially associated with odds of MUO. To the best of our knowledge, this is the first study examining the relationship between DTAC, indicated FRAP, and MUO among adolescents in a Middle Eastern region.

Obesity in children is becoming a growing concern in terms of their health and well-being [1, 2]. So, urgent considerations are required for obesity and its comorbidities in adolescents [42]. Immediate attention in the initial phase of obesity could maintain or improve the MHO condition [43]. It should be underlined that the MHO status of adolescents may shift to MUO condition [44], and such conversion might potentially increase the risk of morbidity and mortality in the future [45]. In the current cross-sectional study, a negative relationship was revealed between total dietary antioxidants and MUO. Thus, it is clinically worthwhile to recommend adolescents increase food options rich in antioxidants such as fruits and vegetables to enhance their diet quality and delay the onset of obesity-related metabolic dysfunction.

Previous investigations indicated that greater consumption of fruits, vegetables, coffee and tea, and nuts was associated with a higher dietary antioxidant intake [46, 47]. In this study, based on the IDF criteria, an inverse relationship was observed between FRAP and MUO among adolescents. When the IDF/HOMA classification was adopted for MUO/MHO, adolescents were classified as MUO/MHO based on abnormal HOMA-IR in combination with IDF criteria. Since some of the MUO cases based on IDF criteria exhibited normal HOMA-IR, they were classified as MHO adolescents based on the second definition. Thus, the non-significant association between DTAC and MUO could be characterized by a small number of cases with MUO based on IDF/HOMA criteria.

Limited data were available on the association between DTAC and metabolic health status in adolescents. A case–control study among 369 Spanish children and adolescents, indicated an inverse relationship between DTAC and obesity-related markers in obese adolescents [48]. Another observational study among the Brazilian obese adolescents reported inverse associations between serum antioxidant micronutrients, such as β-carotene, vitamin E, and retinol with metabolic unhealthy profile [49]. In addition, Ruperez et al. found that retinol concentrations in MUO adolescents were lower than in healthy subjects [25]. Although few studies investigated the association between antioxidant capacity of the diet and metabolic health condition of children or adolescents, some studies have been performed in adults. In a cross-sectional study among 266 young adults, a higher dietary TAC score had a negative relation with some metabolic features such as total cholesterol, abdominal, obesity and oxidized LDL-C concentrations [24]. Furthermore, in a cohort study among 2694 British adults, a higher dietary TAC score was linked to a better glycemic tolerance among individuals with higher BMI [50]. However, another study indicated that antioxidant supplementation would not positively affect metabolic parameters [51]. Contradictory findings from previous studies may be attributable to differences in study design and participants, using different assays for measuring TAC, or various data collection methods. Further investigations are required to confirm whether DTAC is associated with metabolic health status or not.

In the present study, adolescents in the highest category of DTAC score had higher fat and omega-3 fatty acids intake as well as lower carbohydrate intake. Previous studies showed that substitution of carbohydrate with mono- and poly-unsaturated fatty acids, especially omega-3 fatty acids, might reduce the risk of MetS [40]. On the other hand, greater intake of carbohydrate, especially higher intake of refined grains, was associated with lower concentrations of HDL-c, hypertriglyceridemia and hyperinsulinemia among Iranians [39]. In our investigation, energy intake from fat was 30.6% in the highest tertile of DTAC score. In other words, those with the highest fat intake had a relatively moderate-fat diet. While, these participants consumed greater amount of fruit, vegetables, nuts, and dietary fiber. Therefore, one of the possible explanations for the observed association might be related to the amount and quality of macro- and micro-nutrient present in a high DTAC diet.

Although the precise underlying mechanisms have not been identified, some might clarify the observed inverse association between DTAC and MUO. Dietary antioxidants such as polyphenols, carotenoids, and vitamins have been observed to regulate lipid and carbohydrate metabolism and increase insulin sensitivity [52, 53]. Also, the role of oxidative processes has received increasing attention due to their links with obesity complications [54, 55]. Consumption of antioxidant-rich foods might contribute to reduced oxidation by scavenging free radicals and enhancing the availability of TAC in the plasma circulation [56, 57]. In both cross-sectional and randomized intervention trials, a higher intake of TAC has been linked to decreased inflammation and improved circulating antioxidants [23, 58]. Another probable mechanism is that dietary antioxidants could act as anti-inflammatory markers are associated with lower plasma concentrations of CRP [23]. CRP levels are associated with several obesity complications in children [59].

Several strengths of the current study should be underlined. As far as we know, this is the first study that investigated the relationship between the total antioxidant capacity of the diet and MUO in adolescents. Second, during the analysis, several possible confounding were taken into account. Furthermore, blood samples were obtained to assess the metabolic status of participants instead of medical history. However, when interpreting these findings, some limitations must be taken into account. The current study had a cross-sectional design; so, the association between DTAC and MUO cannot be inferred as a causal relation. The causal associations between DTAC and MUO should be confirmed in prospective cohort studies. Furthermore, this point should also be considered that individuals who had higher dietary total antioxidant capacity were more likely to have an overall healthier dietary pattern. In addition, because FFQ is applied to evaluate the dietary intakes, the participants might be misclassified. Finally, the impacts of residual confounders (such as eating habits, puberty, and sleep habits) should also be considered, although several factors have been controlled.

## Conclusion

An inverse association was found between DTAC and MUO, defined by the IDF criteria, among Iranian adolescents. However, based on the HOMA-IR/IDF criteria, no relationship was found between DTAC and MUO. To affirm these findings, more well-designed prospective studies from different countries are warranted.

## Data Availability

Supporting data for this investigation can be available by contacting the supervisor of the research (PS).

## Abbreviations

FFQ: Food frequency questionnaire
OR: Odds ratios
95% CI: 95% Confidence interval
BMI: Body mass index
MHO: Metabolically healthy obesity
MUO: Metabolically unhealthy obesity
IDF: International Diabetes Federation
HOMA-IR: Homeostasis Model Assessment Insulin Resistance
PAQ-A: Physical Activity Questionnaire for Adolescents
SES: Socioeconomic status
DTAC: Dietary total antioxidant capacity
FRAP: Ferric Reducing-Antioxidant Power
ROS: Reactive oxygen species
CRP: C-reactive protein
WHO: World Health Organization
USDA: United States Department of Agriculture
MUFA: Monounsaturated fatty acids
SFA: Saturated fatty acids
SCFA: Short-chain fatty acids
WC: Waist circumference
SBP: Systolic blood pressure
DBP: Diastolic blood pressure
TG: Triglycerides
HDL-c: High density lipoprotein cholesterol
FBG: Fasting blood glucose
MetS: Metabolic syndrome
ANOVA: Analysis of variance
ANCOVA: Analysis of covariance
SPSS: Statistical package for the social sciences
